# Impacts of Diffuse Radiation on Light Use Efficiency across Terrestrial Ecosystems Based on Eddy Covariance Observation in China

**DOI:** 10.1371/journal.pone.0110988

**Published:** 2014-11-13

**Authors:** Kun Huang, Shaoqiang Wang, Lei Zhou, Huimin Wang, Junhui Zhang, Junhua Yan, Liang Zhao, Yanfen Wang, Peili Shi

**Affiliations:** 1 Key Laboratory of Ecosystem Network Observation and Modeling, Institute of Geographic Sciences and Natural Resources Research, Chinese Academy of Sciences, Beijing 100101, China; 2 University of Chinese Academy of Sciences, Beijing 100049, China; 3 Institute of Applied Ecology, Chinese Academy of Sciences, Shenyang 110016, China; 4 South China Botanical Garden, Chinese Academy of Sciences, Guangzhou 510650, China; 5 Northwest Plateau Institute of Biology, Chinese Academy of Sciences, Xining 810001, China; Tennessee State University, United States of America

## Abstract

Ecosystem light use efficiency (LUE) is a key factor of production models for gross primary production (GPP) predictions. Previous studies revealed that ecosystem LUE could be significantly enhanced by an increase on diffuse radiation. Under large spatial heterogeneity and increasing annual diffuse radiation in China, eddy covariance flux data at 6 sites across different ecosystems from 2003 to 2007 were used to investigate the impacts of diffuse radiation indicated by the cloudiness index (*CI*) on ecosystem LUE in grassland and forest ecosystems. Our results showed that the ecosystem LUE at the six sites was significantly correlated with the cloudiness variation (0.24≤R^2^≤0.85), especially at the Changbaishan temperate forest ecosystem (R^2^ = 0.85). Meanwhile, the *CI* values appeared more frequently between 0.8 and 1.0 in two subtropical forest ecosystems (Qianyanzhou and Dinghushan) and were much larger than those in temperate ecosystems. Besides, cloudiness thresholds which were favorable for enhancing ecosystem carbon sequestration existed at the three forest sites, respectively. Our research confirmed that the ecosystem LUE at the six sites in China was positively responsive to the diffuse radiation, and the cloudiness index could be used as an environmental regulator for LUE modeling in regional GPP prediction.

## Introduction

Terrestrial ecosystems play an increasingly important role in global carbon cycle under climate change [1]. Light use efficiency (LUE) was first presented in the context of agricultural ecosystem focusing on the linear relationship between yield and solar irradiance, and gross primary production (GPP) was defined as the overall photosynthetically fixation of carbon per unit space and time [2]. The fact that GPP represents the critical flux component driving the terrestrial ecosystem carbon cycle implies that subtle fluctuations in GPP have substantial implications for future climate warming scenarios [3], [4]. With the quantification terrestrial ecosystem GPP for regions, continents, or the globe, we can gain insight into the feedbacks between the terrestrial biosphere and the atmosphere under global change and climate policy-making facilitation [5], [6]. Still, GPP predictions at regional scale to global scale are a major challenge due to the spatial heterogeneity [7], [8]. Moreover, with the great carbon sequestration potential of the terrestrial ecosystem of China in global carbon budget [9], large uncertainties exist in terrestrial ecosystem GPP simulation in China.

A number of modeling approaches have been developed for regional/global GPP estimations, including ecological process-based models and light use efficiency models driven by remote sensing data [10]. Among all the models, LUE models encompassing the LUE algorithm proposed by [2] may have the highest potential to identify the spatio-temporal dynamics of regional GPP due to the simplicity of concept and availability of remote sensing data [11]. With this method, GPP was defined as product of photosynthetically active radiation (PAR) absorbed by the vegetation canopy and a conversion factor, LUE [2], [12]. Various LUE models have been developed for this purpose, including MODIS GPP algorithm [13], Vegetation Photosynthesis Model (VPM) [14], EC-LUE model [15], Vegetation Index (VI) model [16], C-Fix model [17], Temperature and Greenness Rectangle (TGR) model [18], Temperature and Greenness (TG) model [19] and so on. In order to acquire GPP estimations of high accuracy, the biophysical controls on the ecosystem LUE are significantly important to be fully understood [8], [20]. Recent studies indicated that GPP and LUE were affected by both the quantity and composition of the incoming solar radiation [10], [21]–[23]. With a given value of total incoming radiation, LUE of the entire canopy will increase with the increasing fraction of diffuse radiation (FDR) [23]–[25]. Under cloudy or aerosol-laden skies, incoming radiation was more diffuse and more uniformly distributed in the canopy with a smaller fraction of the canopy that was light saturated [10]. Consequently, canopy photosynthesis was inclined to be more light-use efficient under diffuse sunlight than under direct sunlight condition [10], [21], [23], [24], [26], [27]. Evidences showed that global secondary organic aerosol in the atmosphere will increase by 36% in 2100 [28]. The aerosol influenced the cloud formation, which was the main contributor to the increment on FDR in the atmosphere [29]–[31]. Furthermore, an increasing trend of annual diffuse radiation in China has been proved to be 7.03 MJ.m^−2^.yr^−1^ per decade from 1981 to 2010 [32]. However, few studies on ecosystem GPP predictions took into account effects of the FDR variations of the incoming radiation on LUE based on the LUE models.

Up to now, the eddy covariance (EC) technique provides an alternative way to measure NEE continuously that can be used for GPP calculation by subtracting the modeled ecosystem respiration components [5], [33]–[35]. Multi-sites and continuous eddy covariance (EC) flux and meteorological observation from the ChinaFLUX network provided a valuable tool for GPP and LUE calculation across ecosystems in China [34]. Therefore, in order to reveal the biophysical controls on measured ecosystem LUE for better regional GPP predictions in terrestrial ecosystems of China which is of high spatial heterogeneity, the impact of diffuse radiation resulting from cloud condition on LUE is of growing concern to be characterized by a uniform proxy. Despite the study that effect of cloudiness change on ecosystem LUE and water use efficiency was detected by the clearness index [36], it was difficult to incorporate the clearness index into LUE model for regional GPP estimates due to the specification of the highest interval of solar elevation angle in each grid. Here we employed an cloudiness index algorithm based on simple inputs [13], [22], flux and metrological measurements from six sites of ChinaFLUX encompassing three forest ecosystems and three grassland ecosystems, to address the impact of diffuse radiation on light use efficiency (defined as GPP/PAR) [21], [23], [26]. The objectives of this study are to: (1) illustrate the seasonal dynamics of the cloudiness index and light use efficiency at different sites; (2) address the influence of fraction of diffuse radiation on ecosystem light use efficiency; (3) identify whether the cloudiness index thresholds favorable for enhancing ecosystem carbon sequestration exist or not.

## Materials and Methods

### Sites descriptions and measurements

**Figure 1:**
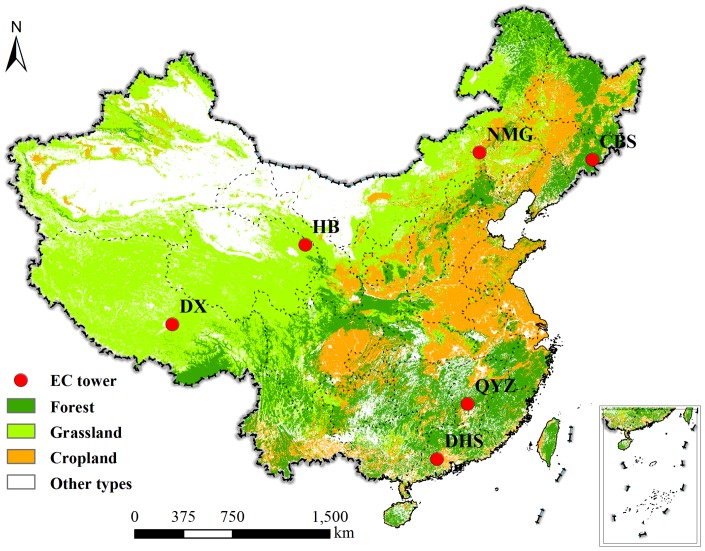
Distribution of the 6 eddy covariance flux sites in China in this study. The background was the MODIS land cover map.

In this study, flux observations were implemented at three forest ecosystems and three grassland ecosystems attached to the Chinese Terrestrial Ecosystem Flux Observational Network (ChinaFLUX). The three forest sites were comprised of the Changbaishan temperate mixed forest (CBS), Qianyanzhou subtropical evergreen needle leaf planted forest (QYZ), and Dinghushan subtropical evergreen broad-leaved forest (DHS). Subject to moosoon-influenced, temperate continental climate, CBS was located in the Jilin province of China, in which growing season ranged from May to September [37]. The QYZ site was located in the subtropical continental monsoon region, in which the mean annual air temperature was 17.9°C [38], [39]. Located in the Guangdong province with a subtropical monsoon humid climate, DHS had a wet season from April to September and dry season from November to March [39]. The three grassland ecosystems were the Inner Mongolia semi-arid *L. chinensis* steppe (NMG) which is C3 grassland, Haibei alpine frigid *P. fruticosa* shrub(HB), and Damxung (DX) alpine meadow-steppe ecosystem with short sparse vegetation(about 10 cm). NMG was located in the Xilin River Basin, Inner Mongolia Autonomous Region of China with a temperate semiarid continental climate. Its growing season lasted from late April to early October [40]. HB was located in the northeast of the Qinhai-Tibet Plateau with a plateau continental climate, which was characterized by lengthy cold winters and very short warm summers. Being situated in a frigid highland, HB receives strong solar radiation, with a mean annual global radiation of up to 6000–7000 MJ.m^−2^ [36], [41]. The DX site was located in the Lhasa City, Tibet, categorized as plateau monsoon climate. Its growing season duration was from May to September. The PAR was usually high, similar to that in alpine meadow area located in eastern Tibetan Plateau and higher than other grassland ecosystems [42]. The locations of six sites were shown in Figure1, and the detailed information of the six sites was provided in Table 1.

**Table 1:** Site descriptions.

Site (ab.)^a^	Changbaishan (CBS)	Qianyanzhou (QYZ)	Dinghushan (DHS)	Haibei (HB)	Inner Mongolia (NMG)	Damxung (DX)
Location	42°24'N	26°45'N	23°10'N	37°40'N	43°32'N	30°51'N
	128°06'E	115°04'E	112°32'E	101°20'E	116°40'E	91°05'E
Elevation(m)	738	102	300	3293	1189	4333
LAI(m^2^m^−2^)	6.1	5.6	4.0	2.8	1.5	1.88
Annual mean precipitation(mm)	600–900	1489	1956	580	350–450	480
Annual mean temperature(°C)	3.6	18.6	21	−1.7	−0.4	1.3
Vegetation type	Mixed forest	Evergreen needle leaf forest	Evergreen broadleaf forest	Alpine frigid shrub	Temperate steppe	Alpine steppe-meadow

a Abbreviation for sites.

Routine meteorological variables were measured simultaneously with the eddy fluxes at each site. Air humidity and air temperature were measured with shielded and aspirated probes (HMP45C, Vaisala, Helsinki, Finland) at different sites. Global radiation and net radiation were recorded with radiometers (CM11 and CNR-1, Kipp & Zonen, Delft, the Netherlands). Photosynthetically active radiation (PAR) above the canopy was measured with a quantum sensor (LI-190Sb, LiCor Inc., USA). All meteorological observations were recorded at 30-min intervals with dataloggers (Model CR10X & CR23X, Campbell Scientific Inc.) [37], [39], [41]–[44].

In the study, we only used data measured during the periods of relatively stable leaf area index (LAI) each year from 2003 to 2007 in order to eliminate the potential effect of changing LAI [36]. The LAI of temperate ecosystems (CBS, NMG, HB and DX) remain stable in the mid-growing season. DHS and QYZ were evergreen forest ecosystems, and their LAI did not vary much with season. Therefore, data of mid growing season (June-August) from all the six flux sites were used to analyze the impact of diffuse radiation on ecosystem light use efficiency and photosynthesis.

### Ethics statement

Three forest ecosystems (CBS, QYZ and DHS) and three grassland ecosystems (NMG, HB and DX) attached to ChinaFLUX were maintained by different institutions of Chinese Academy of Sciences (CAS), respectively. The CBS site was maintained by the Institute of Applied Ecology, CAS; the QYZ site and DX site was maintained by the Institute of Geographic Sciences and Natural Resources Research, CAS; the DHS site was maintained by the South China Botanical Garden, CAS; the NMG site was maintained by University of CAS, and the HB site was maintained by Northwest Plateau Institute of Biology, CAS. All necessary permits were obtained for the described field study. The field study did not involve endangered or protected species. Data will be made available upon request.

### Eddy flux data

Carbon flux data (GPP and NEP) observed at 6 typical sites from 2003 to 2007 across China were applied to in this study (Figure1). The raw 30-min flux data procedure included: (1) 3D coordinate rotation was applied to force the average vertical wind speed to zero and to align the horizontal wind to mean wind direction, (2) flux data was corrected according the variation of air density caused by transfer of heat and water vapor [45], (3)the storage below EC height was corrected for forest sites [46], and (4) the outlier data were filtered and data gaps were filled by using the look-up table method and mean diurnal variation(MDV) [37], [39], [47]. In the end, continuous 30 min flux data was performed.

The flux of net ecosystem CO_2_ exchange (NEE, mg CO_2_ m^−2^ s^−1^) between the ecosystem and the atmosphere was calculated with equation (1), the net ecosystem productivity (NEP) was assigned to –NEE. Negative NEE values denote carbon uptake, while positive values denote carbon source.
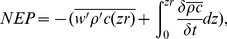
(1)where the first term on right-hand side is the eddy flux for carbon dioxide or water vapor below the height of observation (z_r_), and all advective terms in the mass conservation equation were ignored.

Daily GPP data are partitioned from NEP data measured every 30-min using the eddy covariance technique. GPP was derived from the measured NEP, which was processed using the same method as [36]. Gross primary production (GPP) was calculated employing the following equation:
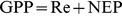
(2)


NEP was obtained directly from the eddy covariance measurement. Ecosystem respiration (Re) of the seven sites was estimated using the Lloyd-Taylor equation (1994) [41], [48]. The nighttime NEP data under turbulent conditions were used to establish Re-temperature response relationship Eq.(3):
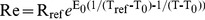
(3)where R_ref_ represents the ecosystem respiration rate at reference temperature (Tref, 10°C); E_0_ is the parameter that determines the temperature sensitivity of ecosystem respiration, and T_0_ is a constant and set as −46.02°C; T is the air temperature or soil temperature(°C). Eq. (3) was also used to estimate daytime Re.

### Calculation of light use efficiency

In this study, LUE (gC.MJ^−1^) was defined as the ratio of daily GPP (gC.m^−2^.d^−1^) to incident PAR (MJ^−1^.m^−2^.d^−1^, using 217 kJ mol^−1^ photons),
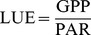
(4)where PAR was directly measured by the in situ meteorological equipment simultaneous with the flux tower observation.

### Cloudiness index

A cloudiness index implemented in CFLUX model was used in our model, since an increase on light use efficiency under overcast conditions at both hourly and daily time steps has been proved in previous studies [22], [49]. The cloudiness index was calculated as [22]:
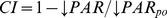
(5)where *CI* is the cloudiness index, ↓PAR is incident PAR(MJd^−1^) from daily observation input, ↓PAR_po_ is potential incident PAR as a derivation of the algorithm of [50]. With the simple inputs of digital elevation model (DEM) data and readily available parameters, the ↓PAR_po_ can be calculated as the global solar radiation at daily time scale in each grid. The spatial resolution of the DEM data was 500 m×500 m, provided by Institute of Geographical Sciences and Natural Resources Research, Chinese Academy of Sciences. More details of the algorithm can be found in the previous literature [50].

The clear sky LUE (LUE_cs_) was specified for each site based on observations of LUE at eddy covariance flux towers. The clear sky LUE was based on the value when ↓PAR/↓PAR_po_ (decreasing cloud cover) approximated 1.0 by a function of LUE under low stress conditions plotted against ↓PAR/↓PAR_po_ [51].

### Statistical analysis

The relationships between different variables were fitted with linear and non-linear equations. All analyses were conducted using the origin package v.8.0 (OriginLab Corporation, Northampton, MA, USA). Statistically significant differences were set with P<0.05 (α = 0.05) unless otherwise stated.

## Results and Discussion

### Seasonal variation of cloudiness index and light use efficiency across ecosystems

**Figure 2:**
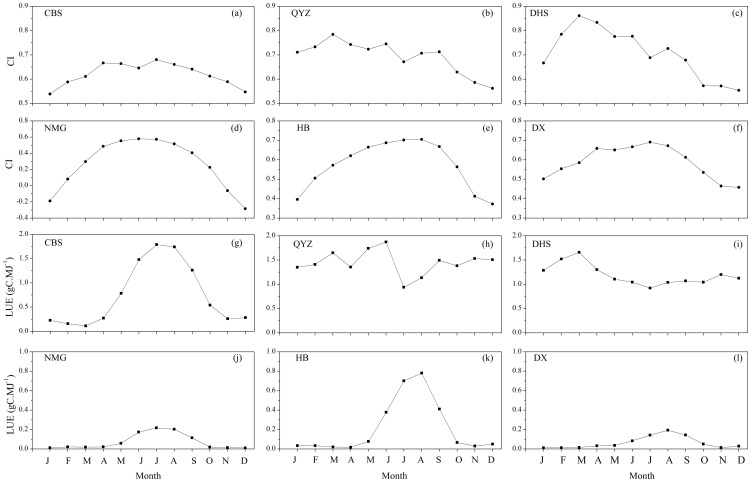
The seasonal variations of monthly mean cloudiness index (*CI*), monthly mean light use efficiency (LUE) from 2003 to 2007 at the five sites.

Figure 2 showed the seasonal variations of the cloudiness index and light use efficiency of the six sites from 2003 to 2006. Mostly, cloudiness index (*CI*) was greater at QYZ and DHS than the other temperate ecosystems (CBS, NMG, HB and DX). The *CI* values of subtropical ecosystems (QYZ and DHS) reached the maximum in March, and were higher during the mid-growing season than the two ends of the year (Figure 2b, c). At the temperate ecosystems, the *CI* values peaked during the mid-growing season (Figure 2a, d, e and f), while the *CI* values of the subtropical ecosystems failed to show substantial variations with the seasonal changes. This indicated that sky conditions of two subtropical ecosystem sites were cloudier than those of four temperate ecosystem sites, and cloudy days were more during the mid-growing seasons at the temperate sites. It was also noted that negative *CI* values were found at NMG site, which was in consistency with meteorological observation that the NMG site received stronger solar radiation during the non-growing season. Meanwhile, the forest ecosystems LUE were significantly higher than grassland ecosystem LUE (Figure 2g, h, i, j, k and l). The LUE at subtropical forest sites (QYZ and DHS) failed to show significantly seasonality, while LUE of the temperate ecosystems (CBS, NMG, HB and DX) peaked during mid-growing season. Furthermore, the ecosystem LUE at QYZ site reached its turning point in July during mid-growing season, presented by a sharp fall resulting from the epidemic summer drought [38]. Among grassland sites, the LUE at HB site exhibited apparently higher values than the other two grassland sites and reached its maximal value in August, whereas the ecosystem LUE at NMG and DX site peaked in July and August, respectively (Figure 2j, k and l).

### Frequency distribution of cloudiness index value across ecosystems

**Figure 3:**
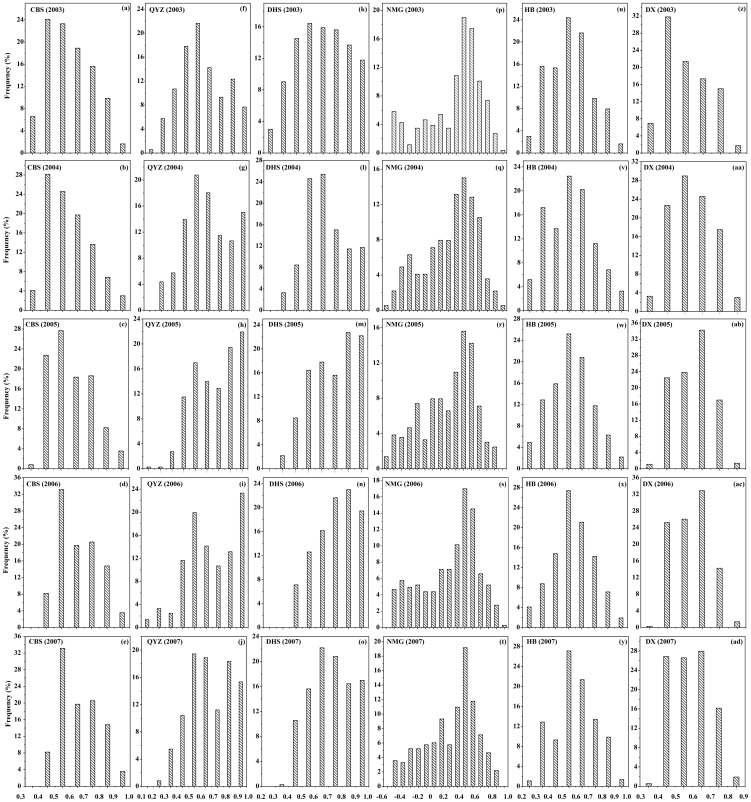
Histograms of the cloudiness index (*CI*) value at the six sites during the mid-growing seasons from 2003 to 2007.

Apart from the seasonal dynamics of sky conditions (Figure 3a–f), the temporal patterns of cloudiness at the six sites in the mid-growing seasons were showed by the frequency distribution of *CI* values (Figure 3). Despite inter-annual variations resulting from climatic variability, common characteristics of the cloudiness pattern were found to be among the six sites. The *CI* values at CBS site occupied the largest frequency around 0.4 in 2003 and 2004(Figure 3a, b), while the *CI* value frequency took the most part around 0.5 from 2005 to 2007(Figure 3c, d and e). The peaks of *CI* value frequency at QYZ site located around 0.5 (Figure 3f, g and j) and 0.9 (Figure 3h, i). The *CI* value frequency at the DHS peaked between 0.5 and 0.7, except for 2005 and 2006, in which the largest frequency occurred around 0.8 in the mid-growing seasons (Figure 3m, n). As to the NMG site, the largest *CI* value frequency occurred between 0.4 and 0.5. Meanwhile, the *CI* frequency peaked around 0.5 at the HB site (Figure3u–z). The *CI* frequency between 0.5 and 0.7 occupied the largest proportion at DX site, except that peaked around 0.4 in 2003(Figure 3z). Overall, the *CI* frequencies occurred between 0.8 and 1.0 in the subtropical forest sites (QYZ and DHS) were much larger than what in the temperate ecosystems (CBS, NMG, HB and DX), which was verified by the report that spatial patterns of annual diffuse radiation in China showed strong regional heterogeneity, lower in the north but higher in the south [32].

### Clear sky light use efficiency in the six ecosystems

The ecosystem LUE was plotted against the ratio of PAR to potential PAR (decreasing cloud cover), and the clear sky LUE (LUE_cs_) was acquired when PAR/PAR_po_ was around 1.0 [51]. LUE_cs_ values were greater at forest sites than those at grassland sites (Table 2). Among the forest sites, the ecosystem LUE_cs_ was greatest at DHS site, intermediated at QYZ site and lowest at CBS site. The LUE_cs_ of forest ecosystem decreased with the degree of latitude. For grassland sites, the LUE_cs_ peaked at the HB site, followed by the DX site and NMG site, respectively. This was verified by the fact that measured ecosystem LUE was higher than the other two grassland ecosystem sites (Figure 2).

**Table 2:** Light use efficiency under clear sky in different ecosystems.

Sites	CBS	QYZ	DHS	NMG	HB	DX
LUE_cs_(gC.MJ^−1^)	0.29	0.425	0.569	0.003	0.013	0.009

### Impacts of diffuse radiation on ecosystem light use efficiency

**Figure 4:**
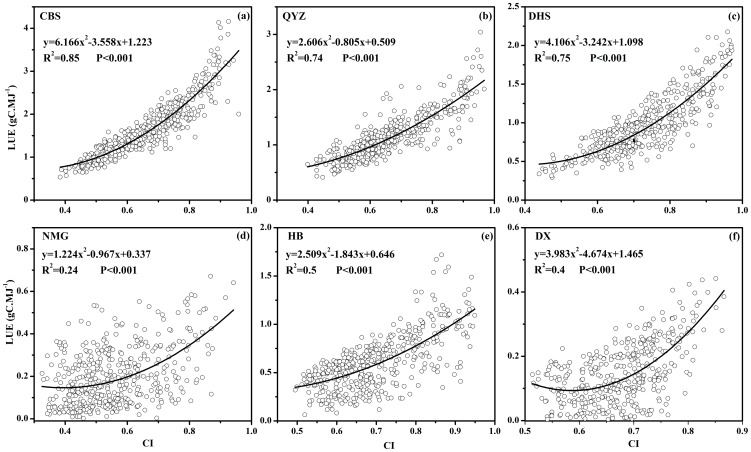
Relationships between LUE and the cloudiness index (*CI*, positive values) during the mid-growing season from 2003 to 2007 at the six sites.

Figure 4 exhibited the interactive responses of ecosystem LUE to the variation in the diffuse radiation fraction of incoming solar radiation (indicated by the cloudiness index) in different ecosystems. At all sites, significantly quadratic regression relationships were found between the ecosystem LUE and *CI* during the mid-growing season. Once the value of *CI* exceeded a certain one, determined by the minimal value (zero) of the first derived function of each quadratic regression function, the ecosystem LUE increased with *CI* dramatically. LUE of forest ecosystem showed more significantly positive relationship with *CI* (R^2^≥0.74), compared with three grassland ecosystem sites (R^2^≤0.5). Also, differences in enhancement on ecosystem LUE induced by the variation of diffuse PAR existed within the ecosystem type across sites. For the forest sites, the ecosystem LUE at CBS site demonstrated stronger increasing trend than the other two subtropical forest sites with the largest correlation coefficient (R^2^ = 0.85) and quadratic term coefficient (6.166)(Figure 4a, 4b and 4c). The ecosystem LUE at NMG site exhibited least increasing potential (R^2^ = 0.24, quadratic term coefficient  = 1.224) with the variation of *CI* among the three grassland ecosystem sites. The expectation that canopy LUE could be enhanced by the diffuse components of solar radiation compared to direct radiation has been reported in previous studies [21], [26], [52]. Under cloudy skies, incoming radiation was more diffuse and more uniformly distributed in the canopy with a smaller fraction of the canopy that was light saturated [10]. Consequently, canopy photosynthesis was inclined to be more light-use efficient under diffuse sunlight than under direct sunlight conditions [10], [21], [23], [24], [26], [27]. In addition, differences in canopy structure density across different ecosystem types was presented to contribute to the increasing rate differences of LUE to fraction of diffuse radiation [36], due to its effective penetration to the lower depths of canopy [32], [53].

### Cloudiness threshold for enhancing forest ecosystem carbon sequestration

**Figure 5:**
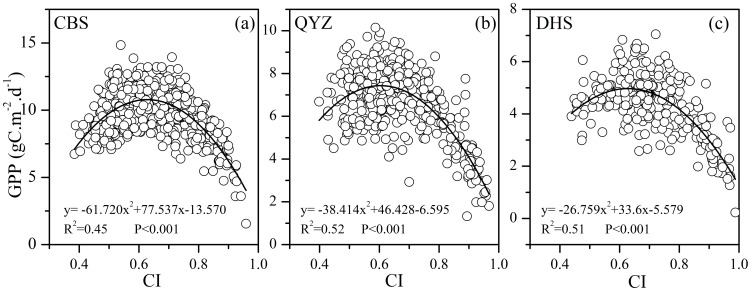
Relationships between ecosystem daily GPP and *CI* during the mid-growing season at three forest ecosystems.

Similar significances of quadratic regression relationships between daily GPP and *CI* during mid-growing season were confirmed at three forest sites (Figure 5). The quadratic regression relationships implied that the ecosystem GPP would peak at a certain value of *CI*, and then decreased with increasingly values of *CI*. Specifically, it was noted that the impact of cloudiness on ecosystem carbon exchange process was also dependent on local thermal, moisture and light conditions [36]. At the beginning stage, the forest ecosystems GPP were in positive association with the *CI*. Instead, the forest ecosystems GPP were gradually restrained by the cloudiness when the value of *CI* exceeded a certain one where the symmetric axis of the parabolic curve regression functions located. This phenomenon could partly be ascribed to the decreasing PAR absorbed by the vegetation canopy, based on the radiation conversion efficiency concept of Monteith (1972) [2]. Consequently, the cloudiness thresholds (Table 3) were calculated by a range that began from the value where the symmetric axis of the parabolic curve regression functions (response of LUE to *CI*) located (Figure 4a, 4b and 4c), and stopped at the point where symmetric axis of the parabolic curve regression functions (response of GPP to *CI*) located (Figure 5). However, the optimal cloudiness index threshold was not available for the three grassland sites because of the poor quadratic relationship between GPP and *CI* (P>0.05). The difference in responses of GPP to the variation of diffuse PAR received by the ecosystem between forest sites and grassland sites was likely to result from the difference in canopy structure [36]. The LAI of forest ecosystem at CBS, QYZ and DHS were higher than those of grassland ecosystem (Table 1). Previous studies reported that LUE and GPP of an ecosystem with low LAI, such as grassland and shrubs, did not increase on cloudy days [54], [55]. This inconsistency was partly attributed to the differences of climate conditions of the studied ecosystems, including light, water and thermal conditions [36].

**Table 3:** Cloudiness thresholds for enhancing forest ecosystem LUE and GPP.

Cloudiness index	CBS	QYZ	DHS
Lower bounds	0.288	0.154	0.394
Upper bounds	0.628	0.604	0.629

## Conclusions

Eddy covariance flux observations from six sites encompassing two ecosystem types and the cloudiness index were used to detect the response of LUE and GPP to diffuse radiation during mid-growing season. Results indicated that (1) cloudiness index (*CI*) was mostly greater at two subtropical forest ecosystem sites (QYZ and DHS) than the other temperate ecosystem sites (CBS, NMG, HB and DX), and LUE in the temperate ecosystem peaked during mid-growing season;(2) LUE under clear sky were greater at forest sites than at grassland sites, and the LUE under clear sky of forest ecosystem decreased with the degree of latitude; (3) significantly quadratic regression relationships were found between the ecosystem LUE and *CI* during the mid-growing season at all sites;(4) cloudiness thresholds favorable for enhancing ecosystem carbon sequestration existed in forest ecosystem sites.

Due to the large regional heterogeneity existing in terrestrial ecosystem of China, more EC flux sites involved in the future are essential to reveal the impacts of diffuse radiation on terrestrial ecosystem LUE in China. Furthermore, the cloudiness index could be incorporated as an environmental regulator into LUE models for regional GPP simulations in China.
